# Using an online survey of healthcare-seeking behaviour to estimate the magnitude and severity of the 2009 H1N1v influenza epidemic in England

**DOI:** 10.1186/1471-2334-11-68

**Published:** 2011-03-16

**Authors:** Ellen Brooks-Pollock, Natasha Tilston, W John Edmunds, Ken TD Eames

**Affiliations:** 1 Centre for Mathematical Modelling of Infectious Diseases, London School of Hygiene and Tropical Medicine, Keppel Street, London WC1E 7HT, UK

## Abstract

**Background:**

During the 2009 H1N1v influenza epidemic, the total number of symptomatic cases was estimated by combining influenza-like illness (ILI) consultations, virological surveillance and assumptions about healthcare-seeking behaviour. Changes in healthcare-seeking behaviour due to changing scientific information, media coverage and public anxiety, were not included in case estimates. The purpose of the study was to improve estimates of the number of symptomatic H1N1v cases and the case fatality rate (CFR) in England by quantifying healthcare-seeking behaviour using an internet-based survey carried out during the course of the 2009 H1N1v influenza epidemic.

**Methods:**

We used an online survey that ran continuously from July 2009 to March 2010 to estimate the proportion of ILI cases that sought healthcare during the 2009 H1N1v influenza epidemic. We used dynamic age- and gender-dependent measures of healthcare-seeking behaviour to re-interpret consultation numbers and estimate the true number of cases of symptomatic ILI in 2009 and the case fatality rate (CFR).

**Results:**

There were significant differences between age groups in healthcare usage. From the start to the end of the epidemic, the percentage of individuals with influenza-like symptoms who sought medical attention decreased from 43% to 32% (p < 0.0001). Adjusting official numbers accordingly, we estimate that there were 1.1 million symptomatic cases in England, over 320,000 (40%) more cases than previously estimated and that the autumn epidemic wave was 45% bigger than previously thought. Combining symptomatic case numbers with reported deaths leads to a reduced overall CFR estimate of 17 deaths per 100,000 cases, with the largest reduction in adults.

**Conclusions:**

Active surveillance of healthcare-seeking behaviour, which can be achieved using novel data collection methods, is vital for providing accurate real-time estimates of epidemic size and disease severity. The differences in healthcare-seeking between different population groups and changes over time have significant implications for estimates of total case numbers and the case fatality rate.

## Background

The severity of influenza, often judged in terms of the Case Fatality Rate (CFR), is a major component in determining the global response to an outbreak [1]. However, severity can be difficult to measure using the CFR because many infections are asymptomatic or mild; furthermore, the estimated CFR depends on the extent to which cases are detected by a surveillance system. During the 2009 H1N1v epidemic in England, the number of symptomatic cases was estimated using GP-based influenza-like illness (ILI) consultation numbers, adjusted for the number of those tested that were virologically confirmed [2,3]. During the H1N1v pandemic, in the absence of a systematic method for quantifying healthcare-seeking behaviour, consultation numbers were scaled-up by assuming that 30% of individuals with ILI sought medical attention at the start of the epidemic, and that this percentage increased to 50% with the launch of the internet- and telephone-based National Pandemic Flu Service (NPFS) in July [2]. These assumptions about healthcare-seeking behaviour clearly play a vital role in determining case estimates. The total number of symptomatic cases was used to estimate an average CFR of 26 deaths per 100,000 cases [2].

Despite the importance of accurately estimating of the number of cases, there is currently no method for systematically assessing healthcare usage. Healthcare-seeking behaviour varies between countries and with individual-level factors such as age, gender or risk group [3,4]. Healthcare-seeking behaviour may also have varied during the course of the 2009 H1N1v epidemic. At the start of the epidemic in June, there were high profile public health campaigns and extensive media coverage, which were associated with increased public anxiety [5,6]. By the start of the autumn wave in September, it was established that the severity of H1N1v was low and there was decreased public interest in the epidemic, potentially affecting GP consultation rates [5,6].

In recent years, internet-based surveillance has been used to provide an alternative measure of influenza activity by collecting information from symptomatic individuals who do not necessarily seek medical attention [7-9]. The UK flusurvey was launched on 16^th ^July 2009 and ran until March 2010 [10]. In this paper, we use the UK flusurvey questions about healthcare usage to provide age- and gender-dependent measures of healthcare-seeking behaviour during the 2009 pandemic in England. In light of this new information, consultation numbers are re-interpreted to provide updated estimates of the number of symptomatic cases and improved estimates of CFR by age group.

## Methods

The baseline numbers used in this analysis were the estimated number of symptomatic H1N1v cases that sought medical attention [2,3]. This was obtained from GP ILI consultation rates and NPFS collections, scaled by an age- and region-dependent proportion of cases that were lab confirmed as H1N1v [3].

### Brief description of the flusurvey

Further details about the UK flusurvey can be found elsewhere [9]. In brief, the UK flusurvey (http://www.flusurvey.org.uk) is an internet-based survey that was launched on 16^th ^July 2009. The survey was publicised via press releases, radio and TV appearances and newspaper articles. There were no restrictions on who could register and volunteers could register throughout the season. The study was approved by the Research Ethics Committee at the London School of Hygiene and Tropical Medicine and conformed to the Helsinki Declaration on ethics of Medical Research. Participants gave their informed consent to take part and were told that anonymised data would be analysed for ILI trends by members of the study team.

The survey consisted of a background questionnaire completed upon enrolment and a weekly symptoms questionnaire (see additional file 1 and [9]).

Volunteers received a weekly email newsletter, which encouraged them to complete the symptoms questionnaire. The symptoms questionnaire asked participants to record their recent ILI and respiratory symptoms, if any. Individuals reporting any symptoms were asked date of symptom onset and whether they had phoned or visited a GP or other medical professional.

### Estimated healthcare usage from the flusurvey

Participants reporting symptoms consistent with the HPA ILI case definition (a fever or high temperature together with two or more influenza-like symptoms of tiredness, headache, runny nose, sore throat, cough, shortness of breath, loss of appetite, aching muscles, diarrhoea or vomiting [11]) were identified as ILI cases. Healthcare usage of these participants was determined using questions from the symptoms questionnaire:

1. Did you phone a medical professional?

• Yes, my GP

• Yes, NHS direct

• Yes, the National Pandemic Flu Service (NPFS)

• Yes, other

• No

2. Did you see a medical professional?

• Yes, my GP

• Yes, hospital A&E

• Yes, I was admitted to hospital

• Yes, other

• No

As national ILI consultation numbers reflect the number of GP consultations by phone, in person and via NPFS, we included all contact with medical services in the analysis. In practice, as few users reported contact with health services other than their GP and the NPFS, we used the proportion that sought no medical attention to avoid double counting for multiple contacts with medical services. Symptom reports were grouped by date and participant gender, age and "at-risk" classification. The mean proportion (and 95% confidence intervals) of ILI cases seeking medical attention, stratified by date, age, gender and risk group, were calculated by bootstrapping. Differences between groups were tested for significance using a non-paired t-test.

To estimate the healthcare-seeking behaviour of a *typical *ILI case during the epidemic, we weighted age- and gender-estimates of healthcare-seeking behaviour by the age distribution of cases.

### Adjusting real-time numbers and estimates of severity

To estimate the number of cases of symptomatic ILI, the number of ILI consultations reported via RCGP and NPFS, stratified by week and age and adjusted by virological positivity rate, was divided by the fraction of flusurvey participants with ILI who sought medical attention, also stratified by age and week (see equation 1).(1)

*l*_*w,a *_is the number of ILI consultations in week *w *for age group *a *estimated from RCGP and NPFS data; *ρ*_*w,a *_is the proportion of ILI consultations that were lab confirmed as H1N1 (in week *w *for age group *a*) estimated through virological surveillance;  is proportion of individuals aged *a *with HPA case definition ILI that sought NO medical attention during weeks [*w *- 1, *w *+ 1], estimated using the flusurvey.

Because of variability in the data, we used a three-week rolling average of healthcare usage. We compared the flusurvey-adjusted case numbers to the current best HPA estimates and real-time estimates reported by Donaldson in 2009 [2]. CFR by age was re-calculated from the ratio of H1N1v deaths to flusurvey-adjusted case numbers for the period up to 8^th ^November 2009 to allow comparison with previous estimates [2].

## Results

### Flusurvey participation

There were 5,738 registered flusurvey participants, approximately two thirds of whom were female; 54% were between 25 and 44 years of age [9]. 21.6% of respondents self-identified as being at higher risk for influenza-related complications (chronic heart disease, diabetes, asthma, chronic lung disease, pregnant, immuno-compromised or other chronic disease).

Volunteers were more likely to report ILI symptoms during their first survey than in subsequent surveys, however retention in the study was over 80% after a participant had completed the survey three times (additional file 2).

Between July and December 2009, the symptoms questionnaire was completed 20,901 times with representation from all UK regions and broadly across all age groups. Individuals reported no symptoms on approximately 60% of occasions. Of the 40% of reports with at least one symptom, 1,522 reports matched the HPA ILI case definition [11].

### Age-associated patterns in healthcare usage

**Figure 1:**
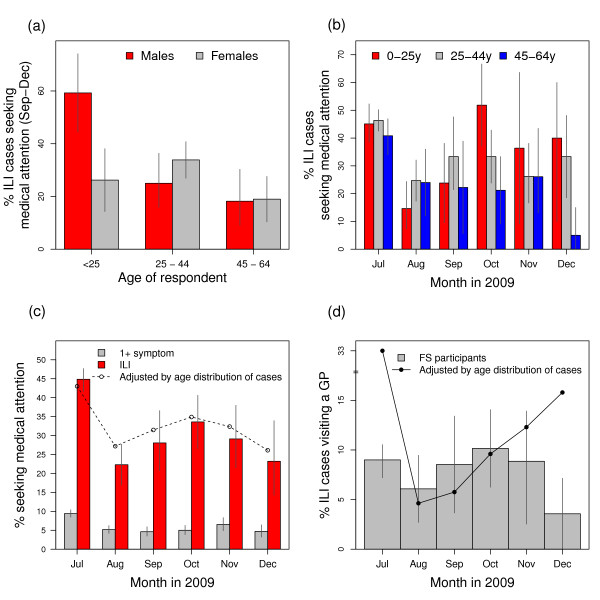
**Estimated healthcare-seeking behaviour during the 2009 H1N1v epidemic in England from the UK flusurvey**. The proportion of UK flusurvey users with HPA case definition H1N1v that sought some form of medical attention between July and December 2009: (a) comparison between age and gender groups during the second wave (September to December 2009); (b) month by month comparison between age groups; (c) healthcare-seeking behaviour of those with case definition ILI compared to that of those reporting other symptoms. The dashed line is the behaviour of a 'typical' ILI case during the epidemic; (d) the proportion of people visiting a GP. The solid line is the behaviour of a 'typical' ILI case during the epidemic. In each panel the solid bars represent the mean and the vertical lines indicate 95% confidence bounds on the mean.

We observed age-dependent healthcare usage during the autumn wave (September to December) of the 2009 H1N1v pandemic (Figure 1a). The greatest difference was between 45 to 64 year-olds, among whom 19% [12%, 25%] of respondents with ILI sought medical attention, and 0 to 24 year-olds, among whom 39% [29%, 49%] of respondents with ILI sought medical attention (p < 0.001). 25 to 44 year-olds showed intermediate behaviour with 32% [26%, 37%] of respondents with ILI seeking medical attention during the autumn wave. The small number of respondents aged over 65 with ILI meant that we were unable to provide detailed estimates of healthcare usage by month, and we found no significant differences between July-August and September-December for this age group.

Overall, we observed that females with ILI were slightly more likely to seek medical attention than males, but the difference was not significant. An exception was males under the age of 25 who were more likely to seek medical attention than females under 25 (Figure 1a). Although there was a significant difference in general healthcare usage between individuals with a self-identified risk factor and those without, we found no significant difference when we compared the healthcare usage of users with ILI.

### Changes in healthcare usage during the epidemic

Healthcare usage changed significantly from month to month during the epidemic (Figures 1b, 1c and 1d). Individuals over 25 years old with ILI decreased their healthcare usage significantly from August onwards: in July 43% [41%, 45%] of ILI cases sought medical attention, whereas between August and December this decreased to 25% [13%, 37%] (p < 0.0001, Figure 1b). In contrast, healthcare usage for individuals with ILI under 25 peaked in October and this is reflected in the overall pattern of healthcare usage. At the peak of the summer wave (week beginning 20^th ^July 2009), 43.1% of ILI cases sought medical attention, whereas at the peak of the autumn wave (week beginning 19^th ^October 2009), 34.5% of ILI cases sought medical attention (Figure 1c). The changes in healthcare usage during the autumn wave were not observed in users with other symptoms not matching the ILI case definition (labelled 1+ symptom in figure 1c), in whom medical attention was sought on an average of 7% [6.4%, 7.5%] of occasions. We observed that in general, the propensity to seek medical attention for ILI increased during the peak of epidemic and decreased during holiday periods.

### Re-estimating case numbers and disease severity

**Figure 2:**
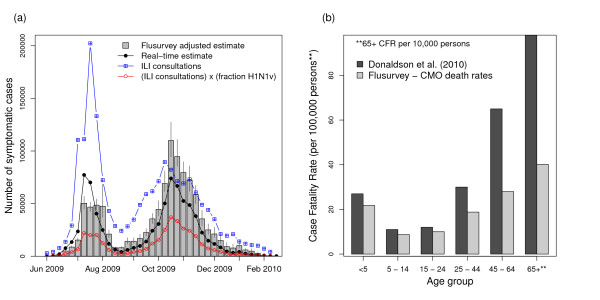
**Re-estimation of epidemic curve and Case Fatality Rate (CFR) accounting for changing healthcare-seeking behaviour behaviour**. a) The flusurvey adjusted epidemic curve (grey bars - the error bars represent 95% CI in healthcare-seeking behaviour) and the HPA real-time estimate (black line with circles). Both estimates are calculated from ILI consultations (blue line with squares) adjusted (red line with dots) using virological surveillance; b) the flusurvey-adjusted Case Fatality Rate (CFR) (light bars) compared to previous estimates [2] (dark bars). The CFR is shown as number of deaths per 100,000 cases for age groups up to 64 years and number of deaths per 10,000 cases for the age group 65 and older (i.e. the values represented by the pair of bars on the right is a factor of 10 greater than the other bars).

Using the healthcare-seeking behaviour estimated from the flusurvey, we estimate that there were 1.1 million symptomatic cases in the UK in 2009 (95% CI = [860,000, 1,600,000]) (Figure 2a). We estimate that the epidemic peaked in the week beginning 19 October 2009 and that 72% of the cases occurred from September 2009 onwards.

Adjusting for the difference in healthcare usage between adults and children shifts the burden of infection towards older age groups. We estimate that the average age of symptomatic ILI cases was 29.3 years, as opposed to an average age of 23.2 years for H1N1v cases seeking medical attention (see additional file 3).

Our increased estimates of the number of ILI cases, compared to previously published figures, result in a 35% overall reduction in CFR to 17 deaths per 100,000 cases. The decrease in CFR was most notable in adults, who were less likely to seek medical attention (Figure 2b). We estimate a CFR of 27 deaths per 100,000 cases in adults aged 45 to 64 years and from 490 deaths per 100,000 cases in adults aged 65 and over.

## Discussion

The analysis presented here - using results from a web-based survey to assess healthcare usage during the 2009 H1N1v influenza epidemic - suggests that individuals with ILI symptoms changed their propensity to seek medical attention during the course of the epidemic. There were significant differences between age groups in healthcare usage of people with ILI, with adults 50% less likely to seek medical attention than children. Higher rates of healthcare usage in children have been previously observed in analysis of NHS direct data prior to the H1N1v pandemic [12].

The healthcare-seeking behaviour of a typical ILI case is affected by the age distribution of cases. In 2009, with the highest attack rates among young people, we found that the large decrease in healthcare usage of ILI cases over 25 years old was tempered by the increase in healthcare usage in those ILI cases under 25 years old. In future seasons, as the average age of cases increases [13] we would expect the healthcare usage of adults to play a more influential role in the observed behaviour.

We estimate that there were 1.1 million symptomatic cases in the UK in 2009 (95% CI = [860,000, 1,600,000]), two thirds more than the real-time estimate [2,3] and 40% more than the current best HPA mid-estimate of 780,000 cases. During the epidemic, it was estimated that 63% of cases occurred after September 2009, but that the epidemic peaked (the greatest number of cases in a single week) during the week of 13^th ^July 2009. The flusurvey-adjusted epidemic profile is more consistent with the pattern of H1N1v deaths, 80% of which occurred during the autumn wave.

The increase in the number of cases results in a 35% overall reduction in CFR from 26 to 17 deaths per 100,000 cases reported previously [2]. In adults 45 and older we estimate a 50% smaller CFR, reducing the CFR from 65 to 27 deaths per 100,000 cases in adults aged 45 to 64 years and from 980 to 490 deaths per 100,000 cases in adults aged 65 and over.

### Strengths and limitations of the surveillance method

A strength of the internet-based approach described here is its ability to assess sentinel surveillance from an alternative perspective; we were able to access people who did not necessarily report their symptoms through any other means.

Telephone-based reporting provides an alterative to internet-based surveillance for community surveillance [14,15]. While telephone-based reporting can control the level participation, a major advantage of web-based implementation is its ability to run throughout the pandemic and to expand to a large number of participants quickly and with little extra cost. In addition to the questionnaire, the flusurvey site provided general information and guidance about influenza and the pandemic to anyone visiting the site. Engagement with the survey was good with a cohort of regular participants, among whom retention was above 80%. We observed that volunteers were more likely to report ILI symptoms during their first survey than in subsequent surveys, suggesting that ILI might have been a stimulus for registering. It is also possible that first-time users could have reported on ILI episodes that had occurred many weeks before they registered, whereas existing users were only asked to report symptoms that had occurred during the past week. In assessing healthcare usage, it is possible that we missed participants who reported their symptoms soon after onset, as they would have had less opportunity to seek healthcare before completing the survey.

Non-probability sampling meant that we recruited several thousand volunteers within a couple of days, although it led to a bias in the age and gender distribution of users. Measured biases in recruitment could be adjusted for using the background questionnaire completed during enrolment. However, as with other active surveillance, it is difficult to quantify how the non-probability sample used differed from the general population in terms of susceptibility to influenza infection and health-care seeking behaviour. The flusurvey, as has been observed with NHS direct [16] and online ILI surveys in other countries [7,17], had an underrepresentation of older people. It is likely that the flusurvey also suffered from the same access issues as NHS direct for individuals with low incomes, from minority ethnic groups or born outside the UK.

## Conclusions

The analysis presented here highlights the benefit of community surveillance for common diseases such as influenza. Standard surveillance is based on those with symptoms seeking healthcare. Thus, if the proportion of those with influenza who seek healthcare is unknown, or changes over time, or varies between age-groups, it is difficult to estimate the number of cases using traditional methods.

Internet surveillance of healthcare usage can be used to complement traditional surveillance by measuring use of healthcare and detecting behavioural changes with minimal delay. For the 2009 H1N1v pandemic, quantifying changes in healthcare usage due to heightened public awareness, media interest, public health campaigns and the temporary National Pandemic Flu Service proved essential for accurately interpreting consultation numbers. Continued surveillance allows us to track changes in behaviour to provide faster and more accurate estimates of incidence and severity, that can ultimately be used to improve the quality of incidence estimates and inform policy decisions during the coming influenza season and future pandemics.

## List of abbreviations

(CFR): Case Fatality Rate; (ILI): Influenza-like illness; (RCGP): Royal College of General Practitioners; (NPFS): National Pandemic Flu Service; (HPA): Health Protection Agency

## Competing interests

The authors declare that they have no competing interests.

## Authors' contributions

EBP performed the analysis and wrote the first draft of the manuscript. KTDE and JE were involved in designing the flusurvey. KTDE, NT, and WJE ran the flusurvey. All authors contributed to the conception, design, interpretation and writing of the manuscript. All authors have read and approved the final manuscript.

## Pre-publication history

The pre-publication history for this paper can be accessed here:

http://www.biomedcentral.com/1471-2334/11/68/prepub

## Supplementary Material

Additional file 1 **Extract from the flusurvey questionnaire, implemented at http://www.flusurvey.org.uk/**. Questions asked as part of the flusurvey implemented during the 2009 pandemic in the UK. Participants were asked the background questions once upon registering and then prompted to answer the symptom questions once a week. Click here for file

Additional file 2 **Participation rates during the flusurvey season**. The horizontal axis denotes the number of times a participant reported and the vertical axis denotes the incremental retention rate for each report number. For example: 41% of participants who reported once reported a second time and of those 72% reported a third time. Click here for file

Additional file 3 **The distribution of infection by age**. The bars show the percentage of people, by age group, who experienced symptomatic influenza-like-illness in England during 2009. Case numbers were calculated using RCGP and NPFS consultation numbers, rates of virological positivity and estimates of healthcare-seeking behaviour taken from the flusurvey (http://www.flusurvey.org.uk). The total number of people in each age group was taken from http://www.census.ac.uk. Click here for file

## References

[B1] "World Health Organization" Pandemic influenza preparedness and response: a WHO guidance document 2009 23741778

[B2] Donaldson LJ Rutter PD Ellis BM Greaves FEC Mytton OT Pebody RG Yardley IE Mortality from pandemic A/H1N1 2009 influenza in England: public health surveillance study BMJ 2009 339 b5213 10.1136/bmj.b5213 20007665 PMC2791802

[B3] Health Protection Agency Weekly epidemiological updates http://www.hpa.org.uk/Topics/InfectiousDiseases/InfectionsAZ/SeasonalInfluenza/EpidemiologicalData/05influsWeeklyinfluenzareportsarchive/

[B4] Royal College of General Practitioners Weekly Returns Service Annual Report 2009 2009

[B5] Rubin GJ Potts HWW Michie S The impact of communications about swine flu (influenza A H1N1v) on public responses to the outbreak: results from 36 national telephone surveys in the UK Health Technol Assess 2010 14 183 266 20630124 10.3310/hta14340-03

[B6] Rubin GJ Amlôt R Page L Wessely S Public perceptions, anxiety, and behaviour change in relation to the swine flu outbreak: cross sectional telephone survey BMJ 2009 339 b2651 10.1136/bmj.b2651 19574308 PMC2714687

[B7] van Noort SP Muehlen M Rebelo de Andrade H Koppeschaar C Lima Lourenço JM Gomes MGM Gripenet: an internet-based system to monitor influenza-like illness uniformly across Europe Euro Surveill 2007 12 E5 6 17991409 10.2807/esm.12.07.00722-en

[B8] Friesema I Koppeschaar C Donker G Dijkstra F van Noort S Smallenburg R van der Hoek W van der Sande M Internet-based monitoring of influenza-like illness in the general population: Experience of five influenza seasons in the Netherlands Vaccine 2009 27 6353 6357 10.1016/j.vaccine.2009.05.042 19840672

[B9] Tilston N Eames K Paolotti D Ealden T Edmunds WJ Internet-based surveillance of Influenza-like-illness in the UK during the 2009 H1N1 influenza pandemic BMC Public Health 2010 10 1 650 10.1186/1471-2458-10-650 20979640 PMC2988734

[B10] The flusurvey website http://www.flusurvey.org.uk/

[B11] "Royal College of General Practitioners", "British Medical Association's General Practitioners Committee" Pandemic influenza: Guidance for GP practices Swine flu H1N1 preparedness 2009

[B12] Bibi M Attwell R Fairhurst R Powell S Variation in the usage of NHS Direct by age, gender and deprivation level Journal of Environmental Health Research 2005 4 63 68

[B13] Bansal S Pourbohloul B Hupert N Grenfell B Meyers LA The Shifting Demographic Landscape of Influenza PLoS ONE 5 2 e9360 10.1371/journal.pone.0009360 20195468 PMC2829076

[B14] Malone JL Madjid M Casscells SW Telephone survey to assess influenza-like illness, United States, 2006 Emerging Infect Dis 2008 14 129 135 10.3201/eid1401.070265 18258092 PMC2600145

[B15] Presanis AM De Angelis D Hagy A Reed C Riley S Cooper BS Finelli L Biedrzycki P Lipsitch M The New York City Swine Flu Investigation Team3¶ The Severity of Pandemic H1N1 Influenza in the United States, from April to July 2009: A Bayesian Analysis PLoS Med 2009 6 e1000207 10.1371/journal.pmed.1000207 19997612 PMC2784967

[B16] Shah SM Cook DG Socio-economic determinants of casualty and NHS Direct use Journal of Public Health 2008 30 75 81 10.1093/pubmed/fdn001 18216300

[B17] Marquet RL Bartelds AI van Noort SP Koppeschaar CE Paget J Schellevis FG van der Zee J Internet-based monitoring of influenza-like illness (ILI) in the general population of the Netherlands during the 2003-2004 influenza season BMC Public Health 2006 6 242 242 10.1186/1471-2458-6-242 17018161 PMC1609118

