# Supplementary material for: Using an online survey of healthcare-seeking behaviour to estimate the magnitude and severity of the 2009 H1N1v influenza epidemic in England
Source: BMC Infect Dis. 2011 Mar 16;11:68. doi: 10.1186/1471-2334-11-68 (PMC3073914; doi:10.1186/1471-2334-11-68)
Supplement: Additional file 1 — Extract from the flusurvey questionnaire, implemented at http://www.flusurvey.org.uk/. Questions asked as part of the flusurvey implemented during the 2009 pandemic in the UK. Participants were asked the background questions once upon registering and then prompted to answer the symptom questions once a week. [file 1471-2334-11-68-S1.PDF]

Additional file for
Using an online survey of healthcare-seeking behaviour to estimate the magnitude and severity of the 2009 H1N1v influenza epidemic in England
Ellen Brooks-Pollock, Natasha Tilston, W. John Edmunds, Ken T. D. Eames

Extract from the flusurvey, implemented at http://www.flusurvey.org.uk

Background questionnaire

Q1. Home postcode:
Q2. Work postcode:
Q3. Gender
- Male
- Female

Q4. Year of birth
Q5. Where do you spend time on a normal day?
- School
- Work
- Home
- Other

Q6. What means of transportation do you use on most days?
Q7. How many colds do you catch each year?
Q8. Did you received a flu vaccine in the last year?
Q9. Why did you choose to receive a flu vaccine?
Q10. Do you have any of the following conditions (tick all that apply)?
- Chronic heart disease
- Diabetes
- Asthma
- Other chronic lung disease
- Pregnant
- Immunocompromised
- Other chronic disease
- None of the above

Q11. Do you smoke?
Q12. How many hours of exercise do you do each week?
Q13. Including you, how many people live in your household?
Q14. Do any of the children in your household go to school/nursery?
Q15. Do you have frequent contact with any of the following (tick all that apply)?
- Children
- Patients
- The elderly
- Large numbers of people
- None of the above

Q16. How did you find out about this survey?

Symptoms questionnaire

Q1. Have you had any of the following symptoms since you last completed the survey (or, for new users, in the last week)?
- Blocked/runny nose
- Cough
- Sore throat
- Headache
- Muscle and or joint pain
- Chest pain
- Stomach ache
- Diarrhoea
- Nausea
- Chills weakness

- Eye irritation
- Fever
- OR no symptoms

Q2. When did the symptoms start?

Q3. Did you have a fever?
- No fever
- 37-39°C
- Greater than 39°C
- Yes, but not measured

Q4. When did your fever start?

Q5. Did your fever come on quickly?
- Yes
- No
- I don't know

Q6. Did you contact a medical professional via the phone or internet (tick all that apply)?
- Yes, my GP
- Yes, NHS direct
- Yes, National Pandemic Flu Service
- Yes, other
- No

Q7. How long after your symptoms appeared did you first contact a medical professional via the phone or internet?

Q8. Did you see a medical professional (tick all that apply)?
- Yes, my GP
- Yes, I was admitted to hospital
- Yes, hospital A&E/outpatient/out-of-hours service
- Yes, other
- No

Q9. How long after your symptoms appeared did you first see a medical professional?

Q10. Did you alter your daily routine?
Q11. How long were you off work/school for?
Q12. Are you still off work/school?
Q13. Did you take any of the following medication because of your symptoms (tick all that apply)?
- Pain killers
- Expectorants
- Antivirals
- I didn't take any drugs

Q14. As far as you know, have you encountered anyone with flu like symptoms in the last week?
