# Supplementary figures and images for: Using an online survey of healthcare-seeking behaviour to estimate the magnitude and severity of the 2009 H1N1v influenza epidemic in England
Source: BMC Infect Dis. 2011 Mar 16;11:68. doi: 10.1186/1471-2334-11-68 (PMC3073914; doi:10.1186/1471-2334-11-68)

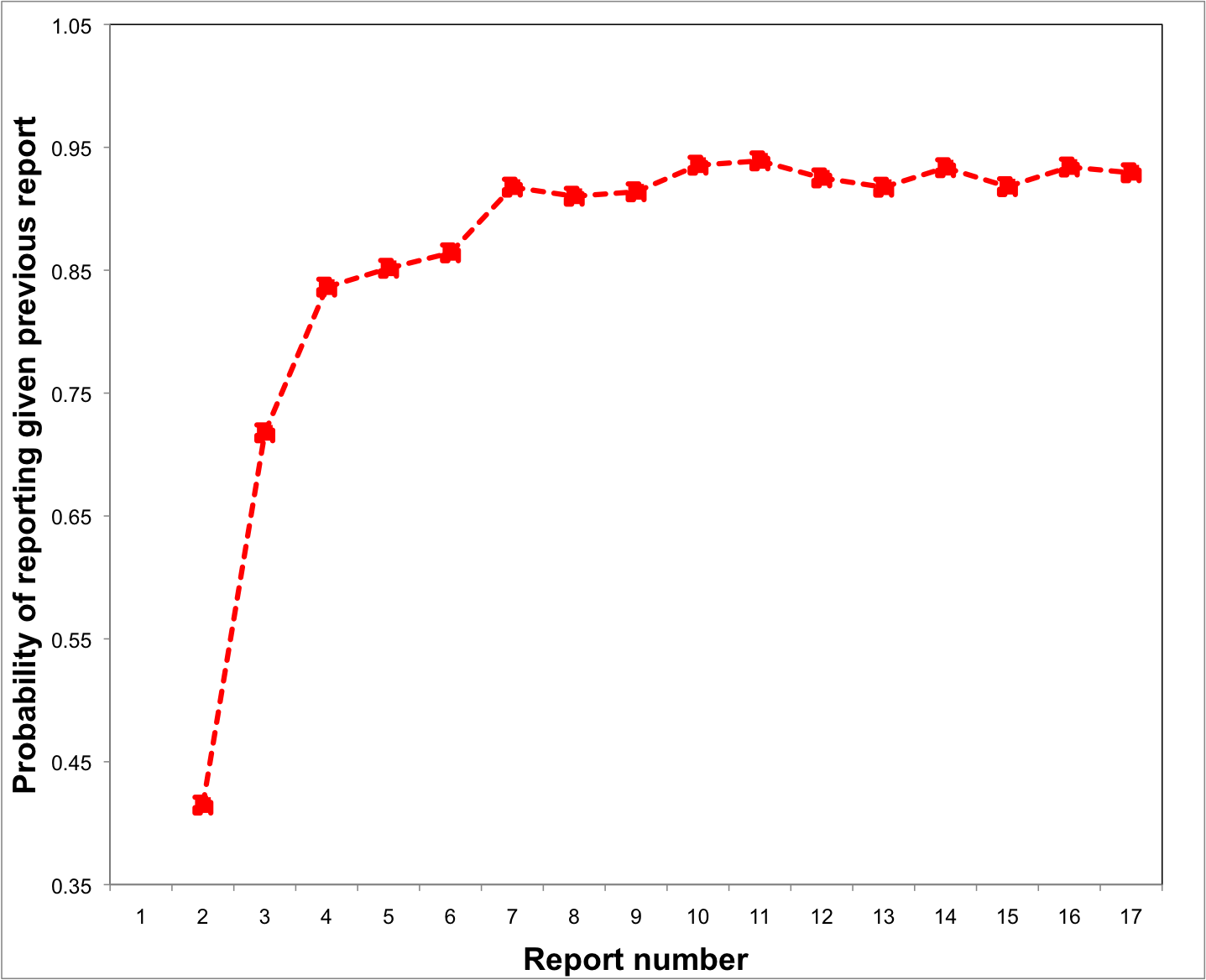

Supplement: Additional file 2 — Participation rates during the flusurvey season. The horizontal axis denotes the number of times a participant reported and the vertical axis denotes the incremental retention rate for each report number. For example: 41% of participants who reported once reported a second time and of those 72% reported a third time. [file 1471-2334-11-68-S2.PNG]

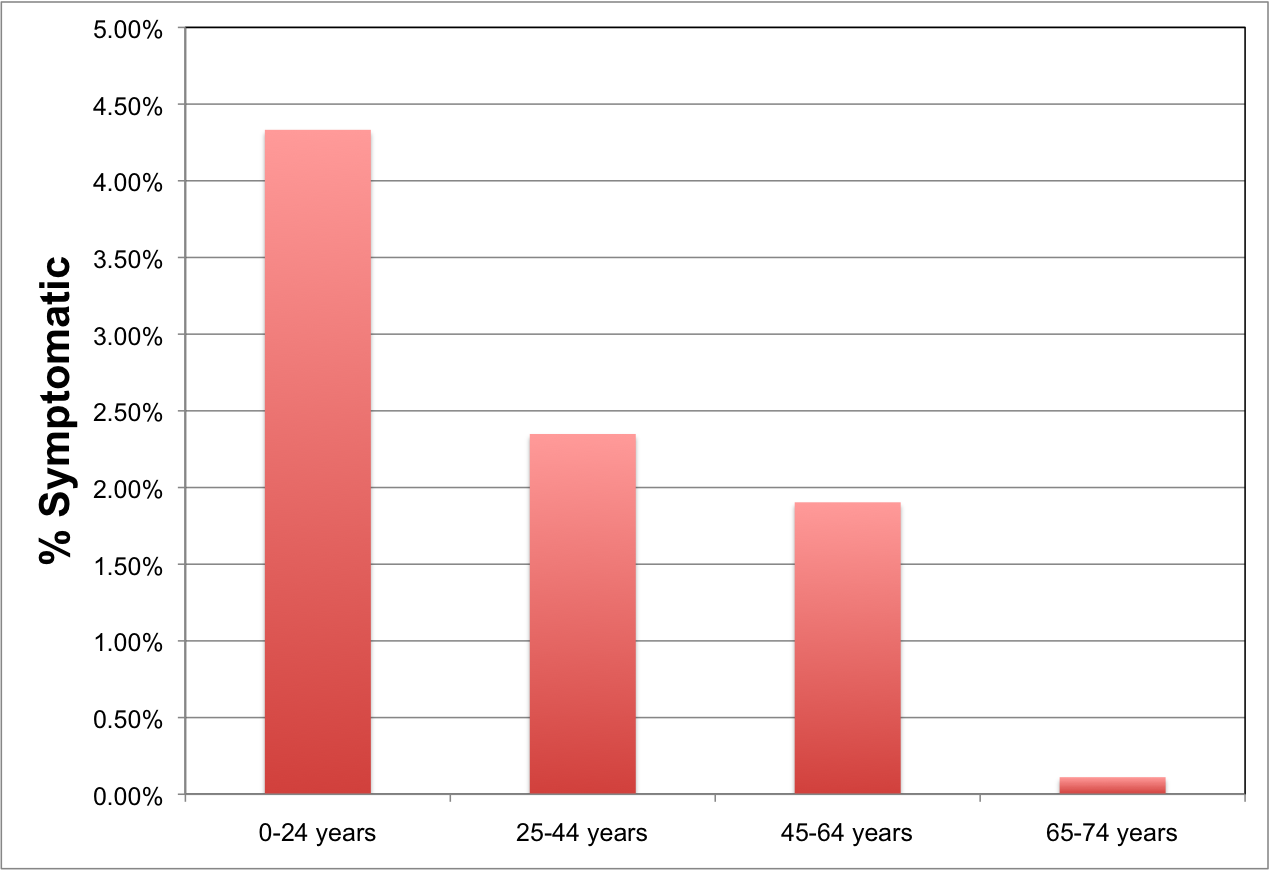

Supplement: Additional file 3 — The distribution of infection by age. The bars show the percentage of people, by age group, who experienced symptomatic influenza-like-illness in England during 2009. Case numbers were calculated using RCGP and NPFS consultation numbers, rates of virological positivity and estimates of healthcare-seeking behaviour taken from the flusurvey (http://www.flusurvey.org.uk). The total number of people in each age group was taken from http://www.census.ac.uk. [file 1471-2334-11-68-S3.PNG]
